# Extracellular Vesicles in Calcific Aortic Valve Disease: From Biomarkers to Drug Delivery Applications

**DOI:** 10.3390/biom15111548

**Published:** 2025-11-04

**Authors:** Alberto Cook-Calvete, Maria Delgado-Marin, Blanca Fernandez-Rodriguez, Carlos Zaragoza, Marta Saura

**Affiliations:** 1 Department of Systems Biology, Faculty of Medicine, Universidad de Alcalá, 28871 Alcalá de Henares, Spain; 2 Instituto Ramón y Cajal de Investigación Sanitaria (IRYCIS), 28034 Madrid, Spain; 3 Centro de Investigación en Red de Enfermedades Cardiovasculares (CIBERCV), 28029 Madrid, Spain; 4 Unidad Mixta de Investigación Cardiovascular, Departamento de Cardiología, Universidad Francisco de Vitoria, Pozuelo de Alarcón, 28223 Madrid, Spain

**Keywords:** calcific aortic valve disease, predictive biomarkers, extracellular vesicles, microRNAs, drug delivery, cardiovascular calcification, valve remodeling, therapeutic targets

## Abstract

Calcific aortic valve disease (CAVD) is a progressive disorder where molecular alterations occur long before visible calcification, making early biomarkers essential. Extracellular vesicles (EVs) have gained attention as stable biomarkers due to their lipid bilayer, which protects proteins, lipids, and RNAs, ensuring reliable detection even in archived samples. This review highlights the role of EVs as biomarkers and delivery tools in CAVD. EVs derived from valvular endothelial, interstitial, and immune cells carry disease-specific signatures, including osteogenic proteins (BMP-2, Annexins), inflammatory miRNAs (miR-30b, miR-122-5p), and lipid mediators. These reflect early pathogenic processes before macroscopic calcification develops. Their presence in minimally invasive samples such as blood, urine, or saliva facilitates diagnosis, while their stability supports long-term monitoring of disease progression and therapeutic response. Advances in purification and single-EV analysis increase specificity, though challenges remain in standardizing methods and distinguishing CAVD-derived EVs from those in atherosclerosis. Beyond diagnostics, engineered EVs show promise as therapeutic carriers. Delivery of anti-calcific miRNAs or combined RNA cargos has reduced calcification and inflammation in preclinical models. Overall, EVs act as molecular mirrors of CAVD, enabling early diagnosis, risk stratification, and novel therapeutic strategies. Yet, clinical translation requires technical refinement and validation of the disease-specific signatures.

## 1. Introduction

Calcific aortic valve disease (CAVD) is increasingly recognized as the main valvular condition among the aging population, with a prevalence approaching 2% in individuals over 65 years old. The disease progresses through fibrotic thickening and calcium buildup within the aortic valve, leading to clinically apparent aortic stenosis (AS) [1]. Without prompt valve replacement, patients with severe AS have a significantly reduced life expectancy, often less than two years [2]. Despite its clinical importance, no effective pharmacological treatments are available at present, partly due to limited understanding of the molecular pathways involved in early aortic sclerosis, the asymptomatic precursor stage. This knowledge gap also hinders the development of early-stage biomarkers that can reliably detect disease onset or progression.

Extracellular vesicles (EVs) have emerged as powerful biomarkers in calcific aortic valve disease (CAVD) due to their ability to encapsulate and transport molecular cargo reflective of disease pathogenesis [3]. These membrane-bound nanoparticles, released by valvular endothelial cells (VECs), interstitial cells (VICs), and infiltrating immune cells, carry a dynamic repertoire of proteins, RNAs (e.g., microRNAs, mRNAs), and lipids that mirror the pathological state of the valve tissue. For instance, EVs derived from calcified aortic valves are enriched in osteogenic proteins such as BMP-2 and Annexins, which drive calcification via activation of Wnt/β-catenin and Notch pathways [4,5]. Proteomic analyses have identified CAVD-specific EV signatures, including WNT5A and amyloid precursor protein, which are absent in healthy valves, highlighting their potential as disease-specific fingerprints [4,6]. Furthermore, EV-associated microRNAs like miR-30b are downregulated in CAVD, and miR-125b leads to depression of osteogenic transcription factors (e.g., RUNX2) and exacerbating calcification [7,8,9]. These findings underscore the role of EVs as molecular snapshots of valve pathology, capturing real-time changes in cellular activity during disease progression.

The lipidomic profile of EVs further enhances their diagnostic utility. Oxidized phospholipids and sphingolipids within EVs correlate with CAVD severity, triggering inflammatory cascades via TLR4/NF-κB signaling in recipient cells [10,11]. Other studies have reported that CD144^+^ endothelial-derived EVs are elevated in acute myocardial infarction, supporting their potential as candidate biomarkers for detecting valvular dysfunction [12]. Similarly, macrophage-derived EVs carrying miR-122-5p are linked to inflammation-driven calcification, offering a mechanistic link between immune activation and osteogenic transformation [13,14]. The stability of EV cargo, particularly RNA, allows for robust biomarker analysis even in archived samples, overcoming limitations of free circulating nucleic acids [15,16].

Despite these advances, challenges remain in standardizing EV isolation and characterizing disease-specific subpopulations. For example, techniques such as ultracentrifugation (UC) and size-exclusion chromatography (SEC) yield variable EV subsets, which affect the reproducibility of cargo profiles [17]. Future studies must prioritize validation of CAVD-specific EV markers (e.g., valve-derived collagen fragments) to distinguish them from atherosclerotic contaminants [18]. Integrating multi-omics approaches—such as proteomics and metabolomics—will further refine EV-based diagnostics, enabling personalized risk stratification and early therapeutic intervention in CAVD [19,20].

## 2. Structural Stability of Extracellular Vesicles in CAVD

The structural stability of EVs represents one of their most valuable attributes for clinical application, particularly in diseases characterized by chronic and progressive tissue remodeling, such as calcification [21]. The defining lipid bilayer envelope of EVs functions as a robust shield that protects encapsulated biomolecules—including microRNAs (miRNAs), messenger RNAs (mRNAs), long non-coding RNAs, and proteins—from degradation in the hostile extracellular environment [22,23,24]. By contrast, as shown in Table 1, the vesicular encapsulation of RNAs significantly prolongs their half-life. Studies have shown that EV-encapsulated miRNAs exhibit up to a tenfold greater stability compared to free-circulating RNAs in serum, retaining integrity even in archived samples that have undergone repeated freeze–thaw cycles [25,26]. This intrinsic protection not only facilitates biomarker discovery but also supports therapeutic applications, since engineered RNA cargos maintain structural fidelity until released in target cells.

The biochemical composition of the EV membrane further strengthens this resilience [27]. Lipid species such as cholesterol, ceramide, phosphatidylserine, and sphingolipids are enriched in EV bilayers, conferring resistance to osmotic stress, oxidative damage, and shear forces experienced during circulation in the cardiovascular system [28]. This composition, similar to the lipid rafts, allows vesicles to preserve their morphology and cargo under mechanical strain such as blood flow turbulence in the aortic valve [29]. This is particularly relevant in CAVD, where the pathological microenvironment is characterized by chronic inflammation, oxidative stress, and the deposition of hydroxyapatite, all of which would typically destabilize synthetic nanoparticles [30]. Nevertheless, EVs maintain their structure, ensuring faithful signal or therapeutic molecule delivery [31].

Pathophysiologically, this structural stability is crucial in CAVD because vesicles secreted by VECs and VICs transport disease-reflective miRNAs, which regulate calcification and osteogenic differentiation [32]. Remarkably, these vesicle-bound miRNAs are detectable in circulation without significant degradation [33,34]. This durability provides a reliable source of disease-associated nucleic acids, supporting their translational value both as biomarkers and therapeutic carries [35,36].

**Table 1:** Comparative Advantages Over Free Circulating Biomarkers.

Parameter	EV-Encapsulated RNA	Free Circulating RNA	Reference
RNase Resistance	High (lipid bilayer protection)	Low (direct exposure)	[25]
Stability in Storage	>6 months at −80 °C	Degrades within weeks	[26]
Signal-to-Noise Ratio	High (enriched cargo)	Low (diluted in biofluid)	[25]
Disease Specificity	Cell-of-origin signatures	Non-specific degradation products	[37]

EVs protect RNA through lipid bilayers, optimizing their use as biomarkers in CAVD [38].

## 3. Extracellular Vesicles Across Biofluids Enhance Diagnostic Accessibility

Another key advantage of EVs is their broad distribution across diverse biological fluids, which significantly enhances diagnostic accessibility in cardiovascular disease [39]. Unlike tissue biopsies, which are invasive and impractical for routine monitoring, EVs can be isolated from minimally invasive sources such as blood, urine, saliva, and even cerebrospinal fluid [40]. Importantly, their encapsulated cargos maintain stability for prolonged periods under storage and handling conditions that would rapidly degrade free-circulating molecules [41]. In blood plasma, for example, EV-associated RNAs remain stable for more than 72 h at 4 °C, whereas free RNAs degrade within hours due to RNase activity [42,43]. This stability facilitates sample handling in clinical and multicenter studies [44].

For CAVD specifically, the capacity to detect and analyze EVs in serum or plasma provides a non-invasive “liquid biopsy” reflecting molecular processes within the aortic valve [45]. Several studies have confirmed that serum-derived EVs from CAVD patients contain calcification-associated miRNAs, including miR-16, miR-24, miR-451, and miR-181a, which remain detectable with minimal degradation even after prolonged storage [43,46]. This consistent preservation enhances diagnostic reliability and enables retrospective analyses in archived biobanks.

The diagnostic accessibility of EVs extends beyond CAVD and finds parallels in other cardiovascular conditions. In myocardial infarction and heart failure, plasma EVs have been used to identify stress- and apoptosis-associated miRNAs [47,48], while in other diseases [49,50], urine-derived EVs have been explored as markers of extracellular matrix degradation [51]. In CAVD, the minimally invasive detection of vesicular cargo opens new opportunities for early diagnosis and risk stratification, which are currently lacking in clinical practice. Traditional imaging modalities such as echocardiography and CT only detect advanced structural changes, whereas EV-based biomarkers may reveal earlier molecular perturbations [52,53]. Indeed, recent studies have demonstrated that systemic EV signatures correlate with the extent of valve calcification and predict disease progression [4,54].

Taken together, the cross-fluid availability and inherent cargo stability of EVs position them as highly promising diagnostic tools in CAVD. They offer a minimally invasive, reproducible, and biologically meaningful window into disease biology, capable of complementing existing imaging-based assessments.

## 4. Extracellular Vesicles as Robust Biomarkers in CAVD

As mentioned, the physical resilience of EVs enables stringent processing in biomarker workflows, overcoming key limitations of traditional circulating biomarkers [55]. High-resolution purification techniques such as density gradient UC and SEC effectively isolate EVs while excluding contaminating proteins and lipoproteins that interfere with downstream RNA analysis [56]. These methods preserve EV integrity and enhance specificity, as demonstrated in studies optimizing SEC for cardiovascular applications [57,58,59]. Recent advancements in dual-mode SEC + UC further improve EV purity by depleting abundant plasma proteins, enabling deeper proteomic and transcriptomic analysis of EV-derived biomarkers [58].

EVs facilitate sensitive detection of disease-specific molecules because their encapsulated RNAs are protected from degradation and enriched in pathological signatures [60]. For example, macrophage-derived EVs carrying miR-122-5p or antiosteogenic miRNAs like miR-30b can be detected at lower concentrations than free RNA via qPCR or sequencing, offering superior signal-to-noise ratios in CAVD diagnostics [33,61]. Multiplexed techniques, such as single-EV analysis, enhance detection by profiling multiple biomarkers simultaneously, revealing heterogeneity in EV subpopulations correlated with disease stages [62].

The stability of EV cargo supports longitudinal monitoring of CAVD progression. For instance, Annexin V^+^-EVs, which reflect calcification activity, can be tracked over time to assess therapeutic response or disease advancement [3,63,64,65]. Emerging technologies like multiphoton microscopy and fluorescence lifetime imaging enable non-invasive, spatiotemporal tracking of EV-mediated processes, such as oxidative stress and matrix remodeling, in preclinical models [66].

Free RNAs are vulnerable to rapid clearance by nucleases and bind inconsistently to carrier proteins (e.g., Ago2), causing variability in measurements. In contrast, EV RNAs reflect parent-cell biology with high fidelity, as demonstrated in CAVD studies where EV miR-30b levels inversely correlate with osteogenic gene expression in valve tissue [67].

These membrane-bound nanoparticles carry a diverse cargo of proteins and lipids that act as disease-specific fingerprints, capturing pathological processes in a minimally invasive manner [54]. As detailed in Table 2, specific EV-associated proteins, such as osteopontin (OPN) and matrix metalloproteinase-9 (MMP-9), along with distinct lipid species including oxidized phospholipids, have been linked to inflammatory and osteogenic signaling pathways in the valve. While these cargos provide valuable insights, their presence alone may not be entirely specific to CAVD, as similar molecules can also be detected in other inflammatory or cardiovascular conditions, emphasizing the need for integrated analysis [33].

**Figure 1:**
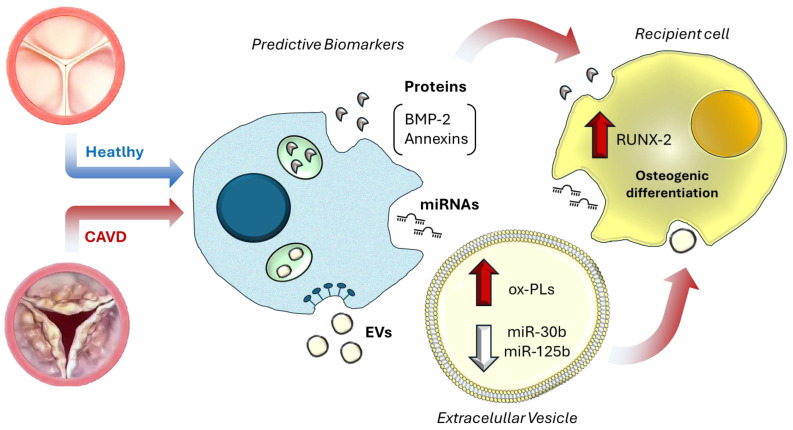
Extracellular vesicle-mediated molecular mirrors in CAVD. In CAVD, extracellular vesicles released from valvular cells carry disease-specific molecular cargo, including oxidized phospholipids (ox-PLs), pro-osteogenic proteins such as bone morphogenetic protein 2 (BMP-2) and Annexin V, and regulatory microRNAs (miR-30b, miR-125b). These molecules can be detected both freely circulating in plasma and encapsulated within EVs, acting as “molecular mirrors” that reflect ongoing pathological processes. Beyond their biomarker potential, EV cargo exerts functional effects on recipient cells, such as upregulation of runt-related transcription factor 2 (RUNX2), thereby promoting osteogenic differentiation and contributing to valve calcification [9].

Building on this, the selective encapsulation of molecular cargo within EVs further enhances their diagnostic potential. As illustrated in Figure 1, proteins such as BMP-2, annexin V and miRNA 30b when enclosed within EVs, are protected from degradation, allowing robust detection in blood samples [63]. By preserving the integrity of bioactive molecules and restricting their release to EVs, these vesicles act as highly informative carriers, capturing subtle cellular alterations in valvular tissue that may otherwise go unnoticed.

Moreover, EVs enriched with inflammation-related miRNAs, such as miR-30b, miR-125b, and miR-122-5p, have been detected in the blood of CAVD patients [4,33]. The structural stability of EVs combined with minimally invasive collection from blood, positions EVs as superior to traditional circulating biomarkers for early CAVD detection [4].

However, beyond their diagnostic utility, EVs also provide valuable prognostic information. Quantitative changes in specific EV subpopulations—for example, elevated CD144+ endothelial-derived EVs or CD14+ monocyte-derived EVs—have been linked to rapid hemodynamic progression and adverse clinical outcomes in CAVD patients [9,68,69]. Moreover, certain EV cargos, including GDF-15 and PON3, offer superior predictive value for major adverse cardiac events compared to conventional plasma biomarkers, highlighting the translational potential of integrating protein and lipid analyses within EVs for comprehensive patient monitoring [4,70,71,72]. Longitudinal tracking of these EV subpopulations and cargos allows dynamic assessment of disease progression and therapeutic response, capturing information that static imaging or conventional biomarkers cannot provide [63,69].

Finally, as described in Table 3, EV-encapsulated miRNAs offer exceptional potential for differentiating stages of CAVD. Specific miRNAs, such as miR-30b, miR-125b, and miR-122-5p, correlate with osteogenic reprogramming and inflammatory activation in VICs and VECs. Because miRNAs often reflect the cellular origin of EVs, their profiles provide precise molecular signatures capable of distinguishing early, intermediate, and advanced disease stages. Combined with the intrinsic stability of EVs and the minimally invasive nature of blood collection, these small RNAs position EVs as highly versatile biomarkers for both early detection and stage-specific assessment of CAVD [4,33].

Comparative studies further show that EV lipidomic and miRNA profiles mirror key pathological processes in the valve: for instance, oxidized phospholipids indicate oxidative stress, while downregulation of miR-30b reflects osteogenic reprogramming of VICs [7,16,73,74]. Emerging technologies, including microfluidic chips and electrochemical biosensors, are enhancing the sensitivity and specificity of EV-based diagnostics, paving the way for point-of-care applications and real-time monitoring of disease mechanisms [75,76].

**Table 3:** Extracellular vesicle-associated microRNAs across stages of CAVD.

CAVD Stage	miRNA	Expression in EVs	Biological Role	Clinical Utility	Refs.
Early Stage (Aortic Sclerosis)	miR-30b	↓ in valve-derived EVs	Inhibits inflammation and osteogenic differentiation	Predicts early calcification risk; inversely correlates with Agatston scores	[77]
	miR-125b	↓ in plasma EVs	Suppresses VIC activation via TRAF6/NF-κB inhibition	Low levels linked to faster hemodynamic progression	[9,33]
	miR-146a	↑ in macrophage EVs	Anti-inflammatory; targets TRAF6/IL-1R to reduce inflammation	Potential therapeutic target	[78,79]
Intermediate Stage (Fibrosis/Calcification)	miR-214	↑ in VIC-derived EVs	Promotes calcification by inhibiting ATF4, an osteoclast activator	Correlates with ECM remodeling and valve stiffness	[80,81]
	miR-122-5p	↑ in VEC-derivedEVs	Drives inflammation via TLR4 signaling in VICs and cardiomyocytes	Elevated in early CAVD plasma EVs; predicts subclinical inflammation	[82,83]
	miR-148a	↓ in circulating EVs	Normally inhibits osteogenic transition via Wnt/β-catenin suppression	Loss correlates with accelerated calcification and AS	[84,85]
Advanced Stage (Severe Stenosis)	miR-21	↑ in platelet EVs	Promotes fibrosis via PTEN suppression and MMP-9 activation	Associated with the need for valve replacement	[86]
	miR-221	↑ in endothelial EVs	Enhances angiogenesis and osteogenesis via p27/CDKN1B inhibition	Linked to adverse post-TAVR outcomes (e.g., paravalvular leaks)	[87,88]
	miR-155	↑ inflammatory EVs	Drives macrophage polarization to pro-calcific (M1) phenotype	Predicts MACE in CAVD patients (e.g., post-AVR heart failure)	[86,89,90]

Summary of selected miRNAs identified in EVs from different cellular origins across CAVD progression stages. Biological roles were assigned based on experimental studies, and clinical utility reflects reported diagnostic, prognostic, or therapeutic relevance. EV origins include VIC—valve interstitial cell; VEC—valve endothelial cell; macrophage EVs; platelet EVs; and circulating/plasma EVs. AS: aortic stenosis; TRAF6: TNF receptor-associated factor 6; NF-κB: nuclear factor kappa-light-chain-enhancer of activated B cells; IL-1R: interleukin-1 receptor; ATF4: activating transcription factor 4; TLR4: toll-like receptor 4; PTEN: phosphatase and tensin homolog; MMP-9: matrix metalloproteinase 9; CDKN1B: cyclin-dependent kinase inhibitor 1B; MACE: major adverse cardiovascular events ↑ indicates enrichment or increased release, whereas ↓ denotes reduction or decreased release.

## 5. Bioengineering of EVs in CAVD

Extracellular vesicles are naturally occurring nanoscale carriers that facilitate intercellular communication through the transport of bioactive molecules such as proteins, peptides, lipids, and nucleic acids [65,91,92]. Recent advances in bioengineering have enabled the functional modification of EVs to enhance their therapeutic potential, particularly in cardiovascular diseases like CAVD [93]. Bioengineering strategies aim to improve cargo specificity, cellular targeting, and biological activity, making EVs an attractive platform for precision therapy [94].

During CAVD, VICs undergo activation, osteogenic differentiation, and extracellular matrix remodeling, leading to progressive calcification and stenosis [95]. To address these pathological processes, EVs can be engineered to carry specific miRNAs that regulate key signaling pathways. For instance, MSC-derived exosomes enriched with miR-146a have been shown to mitigate calcification in vascular smooth muscle cells exposed to advanced glycation end products, downregulating pro-osteogenic markers such as RUNX2 and BMP2 [96]. Similarly, telocyte-derived EVs carrying miR-30b inhibit calcification in VICs via the miR-30b/Runx2/Wnt/β-catenin axis, highlighting the potential of EVs as gene modulators in valvular pathology [33].

Surface engineering further enhances EV specificity and uptake by target cells [97]. Conjugation of valve-targeting peptides, including elastin-binding sequences derived from valvular extracellular matrix, enables selective delivery to aortic valves while minimizing off-target effects [98]. Additionally, modifications such as pH-sensitive fusogenic peptides allow EVs to release their cargo preferentially in acidic microenvironments characteristic of calcifying valves [99,100,101]. This bioengineering approach not only improves therapeutic efficacy but also reduces potential systemic toxicity.

Comparative studies in other cardiovascular diseases provide valuable insights. In atherosclerosis, EVs derived from endothelial or immune cells can either propagate inflammation or confer protective effects depending on their cargo [102,103,104]. Engineering these EVs with anti-inflammatory miRNAs such as miR-145 or miR-21-5p has been shown to stabilize plaques, reduce endothelial activation, and restore autophagic flux [105]. In aortic pathologies, including aneurysms and dissections, MSC-derived EVs carrying miR-146a and angiogenic factors have demonstrated protective effects by attenuating endothelial senescence and promoting vascular repair [106]. These parallels underscore the versatility of EV bioengineering and suggest that strategies effective in one cardiovascular context may be adapted for CAVD.

In addition to nucleic acids, EVs can be loaded with therapeutic peptides and proteins [97,107]. Anti-inflammatory cytokines or matrix metalloproteinase inhibitors can be incorporated to modulate local inflammatory responses and extracellular matrix remodeling [108]. Such multifunctional bioengineered EVs can simultaneously address multiple pathogenic mechanisms, positioning them as a promising regenerative approach for CAVD and related cardiovascular diseases [109].

## 6. EVs as Drug Delivery Systems in CAVD

**Figure 2:**
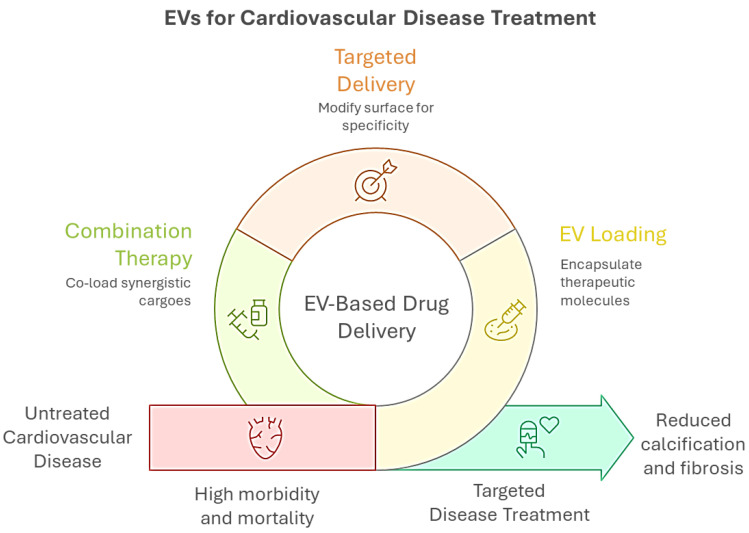
Graphical overview of extracellular vesicle (EV)-based strategies for CVD treatment. EVs can be engineered for drug delivery through surface modification for targeted delivery, encapsulation of therapeutic molecules (EV loading), and co-loading of synergistic agents (combination therapy). These approaches aim to overcome the limitations of untreated CVD, which is associated with high morbidity and mortality, by enabling targeted interventions that reduce calcification and fibrosis, ultimately improving disease outcomes.

EVs, including exosomes and microvesicles, have also emerged as promising drug delivery systems in cardiovascular medicine due to their biological properties (Figure 2). Biological barrier penetration is facilitated by the EV lipid bilayer, which traverses endothelial barriers impermeable to synthetic nanoparticles [109,110].

In the context of CAVD, EVs offer a versatile platform for delivering therapeutic agents directly to diseased valvular tissue, potentially mitigating calcification and fibrosis while minimizing the systemic toxicity associated with conventional pharmacological interventions [108,111]. EVs can be loaded with microRNAs, siRNAs, mRNAs, peptides, or proteins, thereby modulating the activity of VICs, endothelial cells, and inflammatory cells [39,112]. For instance, mesenchymal stromal cell-derived EVs carrying miR-146a have been shown to attenuate osteogenic differentiation and calcification in vascular smooth muscle cells, whereas telocyte-derived EVs enriched with miR-30b inhibit VIC calcification through the Runx2/Wnt/β-catenin pathway [33,113].

For example, EVs loaded with miR-148a (an osteoclast activator) were shown to cross the valvular endothelium in human explants, reprogramming myofibroblasts to suppress hydroxyapatite crystallization [97]. This delivery exploits endogenous trafficking mechanisms, such as clathrin-mediated endocytosis, which is upregulated in CAVD-affected valves [114]. EVs can also transport bioactive proteins and peptides, including anti-inflammatory cytokines or matrix-modulating enzymes that reduce macrophage-driven fibrosis and regulate extracellular matrix remodeling [115,116]. Moreover, surface engineering approaches—for instance, conjugating valve-targeting peptides or elastin-binding motifs derived from the extracellular matrix [117]—enhance the specificity of EV homing to the aortic valve, thereby limiting off-target effects.

Combination therapies represent another promising avenue. Co-loading EVs with synergistic cargoes, such as RUNX2 siRNA (targeting osteogenesis) [118,119] and IL-10 mRNA (anti-inflammatory) [120], has been shown to halt CAVD progression by simultaneously inhibiting calcification and macrophage-driven fibrosis.

The therapeutic potential of EVs as drug delivery systems also extends beyond CAVD to other aortic pathologies, including abdominal aortic aneurysms (AAAs) and aortic dissections [121,122,123]. In AAAs, M2 macrophage-derived EVs delivering miR-221-5p promote anti-inflammatory macrophage polarization, reduce oxidative stress, and preserve vascular smooth muscle cell viability [124,125,126]. Likewise, mesenchymal stem cell-derived EVs attenuate neutrophil extracellular trap-mediated inflammation and elastin degradation, both critical drivers of aneurysm progression [49,127]. In parallel, platelet-derived EVs, which naturally contribute to intercellular vascular signaling, can be engineered to deliver anti-inflammatory molecules or matrix-stabilizing proteins, enhancing tissue repair and reducing rupture risk [128]. Innovative targeting strategies—such as hybrid EVs incorporating monocyte or platelet membranes [129]—further refine localization by directing vesicles specifically to the aneurysmal intraluminal thrombus, thereby improving therapeutic effectiveness [130,131].

Similarly, EV-based approaches are being explored for other aortic conditions, including aortic dissection and coarctation. In dissections, EVs carrying inhibitors of matrix metalloproteinases or collagen-synthesis modulators could stabilize the aortic wall and prevent further tissue degradation [132]. In congenital or acquired coarctation, EVs may deliver agents that modulate endothelial function and smooth muscle cell proliferation [133], counteracting pathological remodeling induced by abnormal shear stress [126]. These studies highlight the versatility of EVs as therapeutic carriers capable of addressing complex, multifactorial vascular pathologies through targeted and multi-modal interventions.

## 7. Clinical Translation of Extracellular Vesicles in CAVD

Despite their promise, several challenges must be addressed for clinical translation of EV-based therapies. Standardization of isolation and characterization techniques is essential to ensure reproducibility and batch-to-batch consistency [134]. Optimizing circulation time and minimizing rapid clearance by the mononuclear phagocyte system are also critical considerations [135]. Additionally, efficient targeting remains a central challenge, requiring ongoing development of ligands or surface modifications that direct EVs to specific tissues [136]. Finally, regulatory pathways for EV-based therapeutics are still evolving, and comprehensive preclinical studies are necessary to evaluate safety, immunogenicity, and long-term effects.

Overall, EVs represent a unique and highly adaptable platform for drug delivery in CAVD and other aortic diseases [137]. Their ability to encapsulate a wide range of molecular cargos, navigate biological barriers, and selectively target diseased tissues positions them at the forefront of emerging cardiovascular therapies [137]. Continued research and optimization of bioengineering strategies will be pivotal in translating these promising preclinical findings into effective clinical interventions capable of addressing the complex pathophysiology of valvular calcification, aneurysm formation, and related vascular disorders.

While EVs carry rich molecular information reflective of CAVD pathogenesis, translating these signatures into clinical biomarkers is challenged by systemic conditions that alter EV release and composition [138]. Chronic kidney disease (CKD), for example, profoundly modifies the EV landscape through uremic toxins, oxidative stress, and impaired renal clearance, resulting in vesicles enriched with pro-inflammatory and matrix-remodeling cargo that may mimic valvular disease signals [139]. Similarly, systemic inflammation and atherosclerosis induce widespread endothelial activation and vesiculation, releasing EVs carrying oxidized phospholipids, annexins, osteopontin, or MMP-9—molecules also implicated in CAVD pathogenesis [138,139]. Because CAVD shares mechanistic pathways with these conditions—including endothelial dysfunction, lipid oxidation, and extracellular matrix remodeling—plasma-derived EVs often represent a composite vascular signal rather than a purely valvular one, particularly in elderly patients or those with CKD, diabetes, or systemic inflammatory conditions [140].

Distinguishing valve-derived EVs from systemic or renal sources is feasible but requires rigorous methodological strategies [140]. Paired valve–plasma studies and multi-omics analyses have identified candidate valve-enriched cargos, such as NOTCH1 and WNT pathway modulators, as well as specific ECM fragments less abundant in atherosclerotic or renal EVs [141]. Incorporating these markers, together with proteomic, lipidomic, and non-coding RNA profiling, can enhance diagnostic specificity [4]. Additionally, stratification by CKD stage, assessment of systemic inflammatory activity (e.g., CRP, IL-6), and immuno-enrichment of valve-endothelial EV subsets may further mitigate confounding signals [142]. From a clinical perspective, EV-based screening could target high-risk populations—older adults (>65 years) with CKD or metabolic syndrome—where early, non-invasive detection would be most impactful. Longitudinal EV profiling, integrated with imaging and clinical metrics, could refine the timing of intervention and improve patient stratification.

Despite these challenges, several human studies are advancing EV-based diagnostics and therapeutics toward clinical translation. The phase I trial NCT05774509 evaluates cardiovascular progenitor cell-derived EVs in non-ischemic dilated cardiomyopathy [143], while the SEAL-HF study (NCT06169540) investigates salivary and plasma EV-associated long non-coding RNAs as biomarkers in acute and chronic heart failure [144]. The EVOC trial (NCT06408961) examines adipose-derived EVs in obesity and cardiometabolic disease, providing mechanistic insights relevant to vascular inflammation and myocardial remodeling [145]. Additional trials, including EASY-AS (NCT04204915) [146] and NCT06002841, explore EVs in early valve replacement and acute respiratory failure, whereas NCT04897841 investigates mesenchymal stem cell therapy in cardiovascular disease, contributing valuable translational data even if not EV-specific [147,148]. Collectively, these studies bridge preclinical discovery and clinical application, establishing safety frameworks, bioprocessing standards, and biomarker validation pipelines relevant to CAVD.

However, critical challenges remain. The mechanistic overlap between CAVD, CKD, and atherosclerosis complicates the identification of truly CAVD-specific EV biomarkers, as systemic vesicles may mimic valvular signatures [138,140,149]. In addition, the absence of unified protocols affects reproducibility, particularly for CAVD-specific markers like NOTCH1 fragments. International initiatives, such as the MISEV guidelines (2023) [150], advocate for orthogonal characterization—combining nanoparticle tracking analysis (NTA) with Western blot—to validate EV isolates. For CAVD, integrating valve-specific markers with universal tetraspanins improves disease specificity. Recent frameworks propose standardized panels (e.g., CD63 + NOTCH1+ EVs) to distinguish pathological valvular signals from atherosclerotic noise [56,151].

Inter-individual variability, comorbidities, and technical heterogeneity in EV isolation and characterization further limit reproducibility and clinical adoption. Isolation variability remains a primary concern, as traditional ultracentrifugation methods induce mechanical damage to EV membranes, leading to cargo degradation and aggregation, while polymer-based precipitation co-isolates contaminants like chylomicrons and lipoproteins [152]. This variability skews quantification of low-abundance CAVD-specific biomarkers, complicating diagnostic accuracy [64]. Emerging solutions leverage microfluidic technologies with immunoaffinity capture, utilizing anti-tetraspanin antibodies (e.g., CD9/CD63) to isolate EVs with >95% purity from plasma [153]. These platforms minimize shear stress and reduce processing time, enhancing yield for downstream CAVD biomarker analysis [26,154,155].

Recent advancements in EV-based diagnostics are reshaping the detection and monitoring landscape of CAVD, particularly by enhancing sensitivity, reproducibility, and applicability at the point of care. A notable development in this area is the adoption of silicon nanowire (SiNW) biosensors, which facilitate ultrasensitive and label-free detection of miRNAs—including those encapsulated in EVs—with remarkable specificity [156,157,158]. Additionally, luminescent SiNW optical biosensors have demonstrated the ability to isolate and quantify EVs—marked with tetraspanin proteins like CD81—with a limit of detection around 2 × 10^5^ small EVs per mL, using minimal sample volume [159,160]. These breakthroughs forgo extensive sample processing and instead favor rapid, high-fidelity analyses.

Complementing these technological strides are efforts to improve standardization and cross-study reproducibility through the use of synthetic EV mimics [157]. These engineered nanoparticles, spiked with calcification-relevant RNA cargos such as RUNX2 siRNA, serve as reference materials to benchmark and calibrate EV isolation kits and detection platforms [161,162]. When used in comparative studies across laboratories, these mimics enable harmonization of protocols and improve the reliability of CAVD biomarker pipelines. Together, these innovations address key bottlenecks in the translational pipeline for EV-based diagnostics—namely, variability in sample processing and limited access to real-time assays—while paving the way for more robust and disease-specific applications in cardiovascular medicine.

## 8. Conclusions

In summary, the combination of valve-specific markers, multi-omics integration, standardized protocols, and carefully designed cohorts will be essential to ensure that EV-derived biomarkers reliably reflect CAVD pathology, enabling early detection, risk stratification, and monitoring of therapeutic interventions.

## Figures and Tables

**Table 2:** Extracellular Vesicle-Associated Proteins and Lipids in CAVD.

Biomarker	Type	EV Source
Proteins	Annexin V	Plasma/Valvular EVs
	BMP-2	Valvular/VIC-derived EVs
	OPN	Plasma/Valvular EVs
	MMP-9	Plasma/Valvular EVs
	GDF-15	Plasma EVs
	PON3	Plasma EVs
	TGF-β1	Valvular/Plasma EVs
	NOTCH1 fragments	Tissue-derived EVs
Lipids	Phosphatidylserine (PS)	Calcifying EVs (plasma/tissue)
	Oxidized phospholipids	Plasma/Lp(a)-associated EVs
	Sphingomyelins/Cholesterol	Valvular/Plasma EVs

The table lists biomarkers detected in EVs derived from plasma, valvular tissue, or valvular interstitial cells (VICs). EV: extracellular vesicle; VIC: valvular interstitial cell; BMP-2: bone morphogenetic protein 2; OPN: osteopontin; MMP-9: matrix metalloproteinase 9; GDF-15: growth differentiation factor 15; PON3: paraoxonase 3; TGF-β1: transforming growth factor β1; NOTCH1: Notch receptor 1; PS: phosphatidylserine; Lp(a): lipoprotein(a).

## Data Availability

No new data were created or analyzed in this study. Data sharing is not applicable to this article.

## Abbreviations

The following abbreviations are used in this manuscript:

AAAs	Abdominal Aortic Aneurysms
Ago2	Argonaute 2
APP	Amyloid Precursor Protein
AS	Aortic Stenosis
ATF4	Activating Transcription Factor 4
AVR	Aortic Valve Replacement
BMP-2	Bone Morphogenetic Protein 2
CAVD	Calcific Aortic Valve Disease
CDKN1B	Cyclin Dependent Kinase Inhibitor 1B
CVD	Cardiovascular Disease
ECM	Extracellular Matrix
EV(s)	Extracellular Vesicle(s)
FLIM	Fluorescence Lifetime Imaging Microscopy
GDF-15	Growth Differentiation Factor 15
IL-1R	Interleukin-1 Receptor
MACE	Major Adverse Cardiac Events
MISEV	Minimal Information for Studies of Extracellular Vesicles
miRNA(s)	microRNA(s)
mRNA(s)	messenger RNA(s)
MMP-9	Matrix Metalloproteinase 9
MPM	Multiphoton Microscopy
MSC(s)	Mesenchymal Stem Cell(s)
NF-κB	Nuclear Factor kappa-light-chain-enhancer of activated B cells
NTA	Nanoparticle Tracking Analysis
OPN	Osteopontin
ox-PL(s)	Oxidized Phospholipid(s)
PON3	Paraoxonase 3
PTEN	Phosphatase and Tensin Homolog
RNA(s)	Ribonucleic Acid(s)
RNase(s)	Ribonuclease(s)
RUNX2	Runt-related Transcription Factor 2
SEC	Size-Exclusion Chromatography
SiNW	Silicon Nanowire
TAVR	Transcatheter Aortic Valve Replacement
TLR4	Toll-like Receptor 4
UC	Ultracentrifugation
VEC(s)	Valvular Endothelial Cell(s)
VIC(s)	Valvular Interstitial Cell(s)
VSMC(s)	Vascular Smooth Muscle Cell(s)
